# Doxorubicin-induced novel circRNA_0004674 facilitates osteosarcoma progression and chemoresistance by upregulating MCL1 through miR-142-5p

**DOI:** 10.1038/s41420-021-00694-8

**Published:** 2021-10-23

**Authors:** Xiao-Long Ma, Tai-Cheng Zhan, Jian-Ping Hu, Chun-Lin Zhang, Kun-Peng Zhu

**Affiliations:** 1 Department of Orthopaedic Surgery, Shanghai Tenth People’s Hospital Affiliated to Tongji University, Shanghai, 200072 PR China; 2 Institute of Bone Tumor Affiliated to Tongji University School of Medicine, Shanghai, 200072 PR China

**Keywords:** Bone cancer, Sarcoma

## Abstract

Accumulating evidence has shown that circular RNA (circRNA) dysregulation is involved in various types of cancer, including osteosarcoma (OS). Nevertheless, the role and mechanism of circRNAs in OS progression and chemoresistance remain elusive. We found that a novel doxorubicin-induced circular RNA, hsa_circ_0004674, screened by whole total transcriptome RNA sequencing in our previous study, was upregulated in OS chemoresistant cell lines and tissues and also connected with patients’ poor prognosis. Circ_0004674 knockdown remarkably suppressed OS cell chemoresistance, proliferation, migration, invasion, OS tumor growth, and enhanced cell cycle arrest and apoptosis in vitro and in vivo through control the expression of the antiapoptotic protein MCL1, a member of the Bcl-2 gene family. Further online bioinformatics analysis revealed that miR-142-5p had potential binding sites that can bind circ_0004674 and the 3′UTR of MCL1 mRNA. Moreover, the expression and function of miR-142-5p were conversely correlated with circ_0004674 in vitro. RIP, pull-down, luciferase assay, and RNA FISH demonstrated that circ_0004674 could compete with MCL1 for miR-142-5p binding to counteract miR-142-5p-mediated repression of MCL1 at the post-transcriptional level. To sum up, our study sheds light on the critical role of the oncogenic circ_0004674/miR-142-5p/MCL1 axis in OS progression and chemoresistance, providing a novel potential target for OS therapy.

## Introduction

Osteosarcoma (OS), the most common primary malignant tumor of bone with peak incidence in children and adolescents, is highly invasive and tends to metastasize to the lung [1, 2]. Chemotherapy combined with complete surgical resection has been the standard treatment for most OS patients with a significantly improved 5-year disease-free survival rate to 70–80% [3]. But the survival rate of patients who suffer from chemoresistance or lung metastasis has declined to less than 20% [4, 5]. Therefore, it is essential to expand the molecular understanding of tumorigenesis and progression of OS to provide novel biomarkers or treatment targets for this refractory disease [6, 7].

With the development of deep-sequencing technology, many novel noncoding RNAs, including circular RNAs (circRNAs), have been identified and found to act a pivotal part in diverse cellular physiological and pathological processes [8, 9]. Unlike its homologous linear transcript, the characteristic of circRNA is that it has a closed-loop structure without a 3′ polyadenylate tail or 5′ cap, making it highly tolerant to RNase R [10]. In addition, many reports have demonstrated that circRNAs can regulate the expression of numerous genes at the pre-, transcriptional, and post-transcriptional levels through interacting with DNA, RNA or protein [8, 11]. Lately, some circRNAs have been discovered to take part in the progression of OS, such as circMYO10 [12], circRNA LRP6 [13], and circ-ITCH [14]. However, the function and related mechanism of action of specific circRNAs in OS progression and chemoresistance have not been clearly illustrated.

In our previous study, we firstly identified the comprehensive circRNA expression profile of three paired chemoresistant and chemosensitive osteosarcoma cell lines (MG63/DXR vs. MG63, KH-OS/DXR vs. KH-OS, U2-OS/DXR vs. U2-OS) and had seeked out 80 differentially expressed circRNAs (57-upregulation, 23-downregulation) [15]. In the current study, we focused on one of the 80 screened circRNAs, hsa_circ_0004674 (chr7:87593948-87607727, 13779 bp), which was the most upregulated, with a 17-fold change in chemoresistant OS cell lines. We first detected the circ_0004674 expression in OS tissues and cells to explore its underlying clinical significance. Then, we determined the vital role of circ_0004674 in OS cell proliferation, chemoresistance, cell cycle, apoptosis, migration, and invasion in vitro and vivo. We also found that circ_0004674 could act as a competitive endogenous RNA (ceRNA) to sponge miR-142-5p to further lead to OS progression and chemoresistance by upregulating the expression of the antiapoptotic protein MCL1, a member of the Bcl-2 family, and circ_0004674 may be a therapeutic target of OS.

## Results

### A novel circRNA, circ_0004674, was upregulated in chemoresistant OS tissues and cell lines and correlated with poor prognosis

**Fig. 1:**
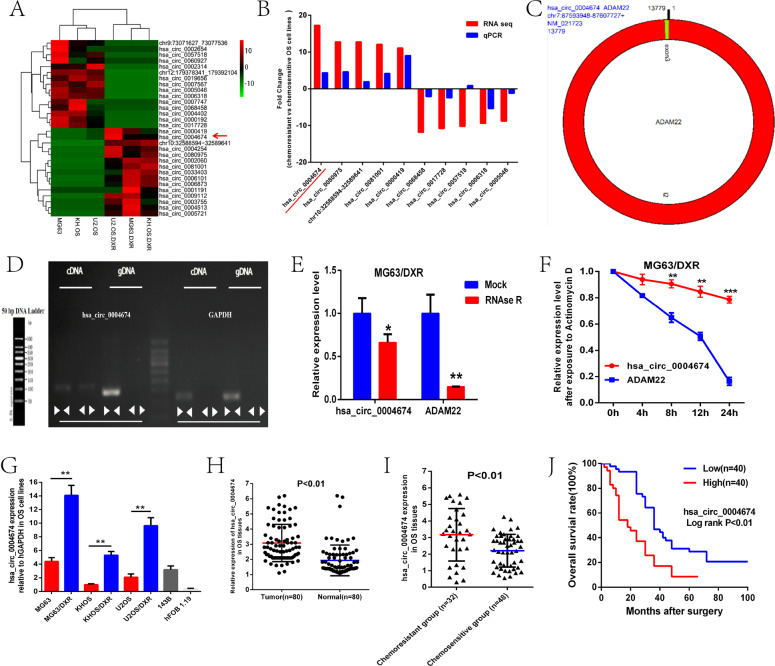
A novel doxorubicin-induced circRNA, circ_0004674, was upregulated in chemoresistant OS tissues and cell lines and correlated with poor prognosis. **A** Heat map showing the top 15 upregulated and downregulated differentially expressed circRNAs screened by RNA sequencing in three paired chemoresistant and chemosensitive OS cell lines. Of them, hsa_circ_0004674 was the most upregulated circRNA with a 17-fold change in the chemoresistant OS cell lines compared to the control. **B** Ten circRNAs (five upregulated and five downregulated) were randomly selected and validated by qRT-PCR. Nine of them, including circ_0004674, were consistent with those of RNA sequencing. **C** CircBase and circPrimer showed that circ_0004674 was derived from intron-exonic back splicing of its homologous ADAM22 gene. **D** Divergent and convergent primers were designed to amplify circ_0004674. Gel electrophoresis showed that divergent primers could produce circ_0004674 in cDNA but not in gDNA. **E, F** RNase R and actinomycin D treatment had no significant impact on the stability of circ_0004674 but had significant impact on the stability of linear ADAM22. **G** Circ_0004674 was significantly upregulated in the three chemoresistant OS cell lines, another OS cell line,143B compared to and the normal osteoblast cell line, hFOB1.19. **H** Circ_0004674 was significantly upregulated in OS tissues than that of paracancerous tissues (3.1 ± 1.2 vs. 1.8 ± 0.8). **I** Circ_0004674 expression was higher in the chemoresistant group than that in the chemosensitive group (3.2 ± 1.5 vs. 2.3 ± 1.1). **J** OS patients with higher expressions of circ_0004674 had less survival time than those with lower expression(20.2 ± 2.8 vs. 36.4 ± 3.6 months). *P < 0.05, **P < 0.01.

In the previous study of our team, we have found a novel circRNA, hsa_circ_0004674, which was the most upregulated circRNA in chemoresistant OS cell lines compared with control cell lines through total transcriptome RNA-seq screening and qPCR verification [15, 16] (Fig. 1A, B). Based on information from the circbase database and circPrimer, we found that circ_0004674 derived from intron-exonic back splicing of its homologous ADAM22 gene which was located on chr7 with a length of 13779 bp (Fig. 1C). Meanwhile, divergent and convergent primers were designed to amplify circ_0004674, and gel electrophoresis experiment showed that circ_0004674 was amplified only by divergent primers in cDNA, not in gDNA (Fig. 1D). RNase R and actinomycin D treatment, that can inhibit polymerase II transcription and block mRNA biogenesis, were given to examine the characteristics of circ_0004674. Treatment with RNase R and actinomycin D had no significant impact on the stability of circ_0004674 but had an obvious impact on the stability of linear ADAM22 (Fig. 1E, F), indicating that circ_0004674 is a true circular molecule.

To identify whether circ_0004674 plays a vital role in OS tumorigenesis, we firstly tested circ_0004674 expression in three paired chemoresistant and chemosensitive OS cell lines, a human OS cell line 143B and a normal osteoblast cell line hFOB1.19, using qRT-PCR. And results showed that circ_0004674 was strikingly increased in chemoresistant OS cells compared with that in hFOB1.19 cells (Fig. 1G). Then, the circ_0004674 expression was examined in 80 paired OS and paracancerous tissues, and the results showed that circ_0004674 was also strikingly increased in OS tissues compared with that in paracancerous tissues (Fig. 1H). We then classified the 80 OS patients into the chemoresistant group and chemosensitive group in light of their clinical Huvos scores and found that the expression of circ_0004674 was higher in the chemoresistant group than that in the control group (Fig. 1I). In addition, K-M survival analysis revealed that OS patients with higher circ_0004674 expression had less survival time than those with lower circ_0004674 expression, which indicated that circ_0004674 was related to OS tumorigenesis and progression with potential clinical significance (Fig. 1J).

### Circ_0004674 promoted OS cell chemoresistance, cell cycle arrest, apoptosis, migration, invasion, and tumor growth in vitro and in vivo

**Fig. 2:**
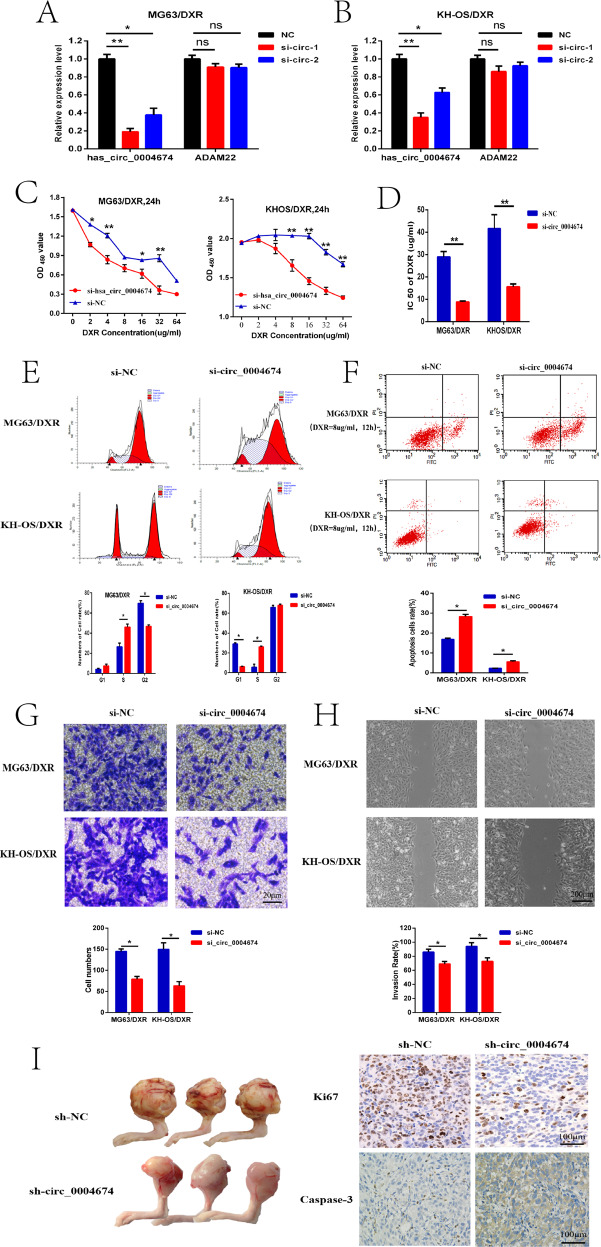
Circ_0004674 promoted OS cell chemoresistance, cell cycle arrest, apoptosis, migration, invasion, and tumor growth in vitro and in vivo. **A, B** qRT-PCR analysis was performed to confirm the effect of siRNAs on circ_0004674 knockdown in MG63/DXR (or KH-OS/DXR) cells. **C, D** CCK-8 assays showed that cell resistance to doxorubicin in the si-circ_0004674 group (MG63/DXR or KH-OS/DXR) was evidently decrease than that in the si-NC group. **E, F** Flow cytometry demonstrated that the rates of cell cycle arrest and apoptosis (after exposed to doxorubicin) in the si-circ_0004674 group (MG63/DXR or KH-OS/DXR) were evidently increase than those in the control group. **G** Transwell assays showed that the capacity of cell invasion after transfection was greatly weakened in the si-circ_0004674 group compared with that in the control group. **H** Wound healing indicated that the capacity of cell migration after transfection was also weakened in the si-circ_0004674 group compared with that in the control group. **I** Tumor xenografts showed that the volume and weights of the formed tumors in the proximal tibia were smaller in the sh-circ_0004674 group than in the sh-NC group. Lower activity of ki-67 and higher activity of caspase3 measured by IHC were detected in the tumor tissues of sh-circ_0004674 group than in the sh-NC group. *P < 0.05, **P < 0.01.

To further explore the biological significance of circ_0004674 on OS chemoresistance and progression, gain- and loss-of-function studies were performed. Because the length of circ_0004674 (13779 bp) was too long and it was difficult to reconstruct its circular structure in cells without affecting the expression of its homologous ADAM22 gene, we constructed transfected cell lines with siRNAs (or sh-RNA) designed for the junction site to knockdown circ_0004674 expression in MG63/DXR (or KH-OS/DXR) cells, which was confirmed by qRT-PCR (Fig. 2A, B). Further CCK-8 assays showed that cell resistance to doxorubicin in the si-circ_0004674 group were evidently decrease than that in the si-NC group (Fig. 2C, D). In addition, flow cytometry further demonstrated that the rates of cell cycle arrest and apoptosis in the si-circ_0004674 group were evidently increase than those in the control group (Fig. 2E, F). Likewise, the transwell assays and wound healing assays showed that the ability of invasion and migration was greatly weakened in si-circ_0004674 group compared with those in the control group (Fig. 2G, H). Furthermore, tumor xenografts showed that the volume and weights of the formed tumors in the proximal tibia were smaller in sh-circ_0004674 group than in sh-NC group. Further IHC assays also showed that the cell proliferation activity performed by Ki-67, was lower in sh-circ_0004674 group than the controlled, contrary to the activity of apoptotic protein caspase3 (Fig. 2I). These results indicated that circ_0004674 promoted OS chemoresistance and progression in vitro and in vivo.

### Circ_0004674 could sponge miR-142-5p to function as a ceRNA at the post-transcriptional level in OS progression

**Fig. 3:**
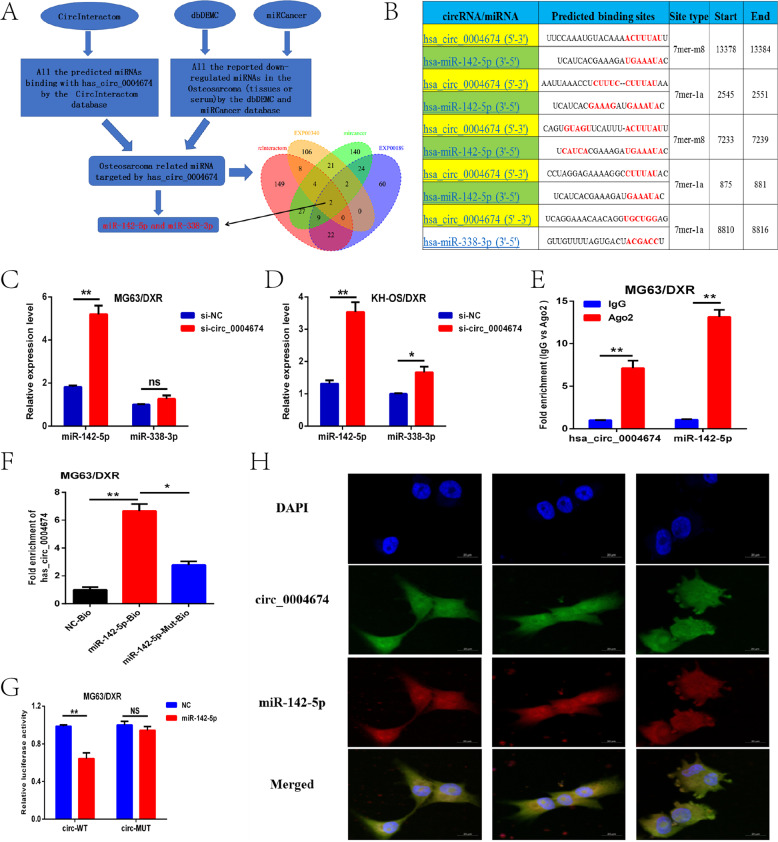
Circ_0004674 could sponge miR-142-5p to function as a ceRNA at the post-transcriptional level in OS progression. **A** Two miRNAs, hsa-miR-142-5p and hsa-miR-338-3p, which had potential binding sites for circ_0004674 and were simultaneously downregulated in OS tissues and serum, were identified based on three online databases (CircInteractome, dbDEMC and miRCancer). **B** Predicted potential binding sites for circ_0004674 and miR-142-5p or miR-338-3p. **C, D** miR-142-5p expression was significantly increased when circ_0004674 was knocked down in the MG63/DXR (or KH-OS/DXR) cell lines, whereas miR-338-3p expression did not obviously change in the two cell lines. **E** RIP assays showed that circ_0004674 and miR-142-5p could both bind to the Ago2 protein. **F** Biotin-coupled probe pull-down assay showed that using the biotin-labeled miR-142-5p (miR-142-5p-bio) probe increased the expression of circ_0004674 compared with the control (NC-bio) or miR-142-5p-Mut-Bio probes. **G** Luciferase activity assay showed that circ_0004674 could combine with miR-142-5p. **H** Subcellular localization by RNA FISH showed that both circ_0004674 and miR-142-5p were mainly colocalized in the cytoplasm. *P < 0.05, **P < 0.01.

Circular RNA acting as a sponge molecule is one of its vital functions at the post-transcriptional level because of its many potential miRNA binding sites [8]. Then, we speculated that circ_0004674 could sponge some important OS-related or chemoresistance-related miRNAs that are involved in the regulation of OS progression and chemoresistance. By using online bioinformatics databases (CircInteractome), we found 221 predicted miRNAs that may interact with circ_0004674. Considering the negative correlation between circRNA and miRNA in the ceRNA mechanism, OS-related downregulated miRNAs were collected from the dbDEMC 2.0 and miRCancer databases. We found that 119 miRNAs and 227 miRNAs were downregulated in OS tissues from the dataset of EXP00189 from dbDEMC and miRCancer, respectively. In addition, we found that 143 miRNAs were downregulated in the serum of OS patients compared to that of normally controlled participants from the dataset of EXP00340 from dbDEMC. There were only two miRNAs, namely, hsa-miR-142-5p, and hsa-miR-338-3p, that had potential binding sites for circ_0004674 according to the comprehensive analysis results of the four datasets and which were simultaneously downregulated in the OS tissues and sera (Fig. 3A). As shown in Fig. 3B, there were four potential binding sites between circ_0004674 and miR-142-5p, but only one binding site between circ_0004674 and miR-338-3p. Furthermore, miR-142-5p expression was significantly increased when circ_0004674 was knocked down in the MG63/DXR and KH-OS/DXR cell lines, whereas miR-338-3p expression did not obviously change in the two cell lines exposed to the same conditions (Fig. 3C, D). Therefore, we speculated that miR-142-5p may be the most important miRNA for circ_0004674 in OS progression.

Further RIP assays and biotin-coupled probe pull-down assays were employed to explore whether circ_0004674 could sponge miR-142-5p. As shown in Fig. 3E, circ_0004674 and miR-142-5p were more abundant in the Ago2 pellet than in the IgG pellet in MG63/DXR cells. Moreover, a biotin-coupled probe pull-down assay revealed that using a biotin-labeled miR-142-5p (miR-142-5p-bio) probe increased circ_0004674 expression compared with the control (NC-bio) or miR-142-5p–mut-bio probes (Fig. 3F). To verify that miR-142-5p was a direct target of circ_0004674 and the predicted binding sites between them, we constructed luciferase reporter plasmids containing wild type (WT) circ_0004674 and mutant-type (MUT) circ_0004674. As predicted, co-transfection of luciferase reporter plasmid that containing WT-circ_0004674 with the miR-142-5p mimics into MG63/DXR cells led to decreased reporter activity (Fig. 3G). Whereafter, RNA FISH (RNA fluorescence in situ hybridization) was implemented to confirm the interactions between circ_0004674 and miR-142-5p in terms of subcellular localization, and the results showed that both of them were mainly colocalized in the cytoplasm (Fig. 3H). All the above results suggested that circ_0004674 could sponge miR-142-5p to function as a ceRNA at the post-transcriptional level in OS progression.

### MiR-142-5p suppressed OS chemoresistance by targeting MCL1

**Fig. 4:**
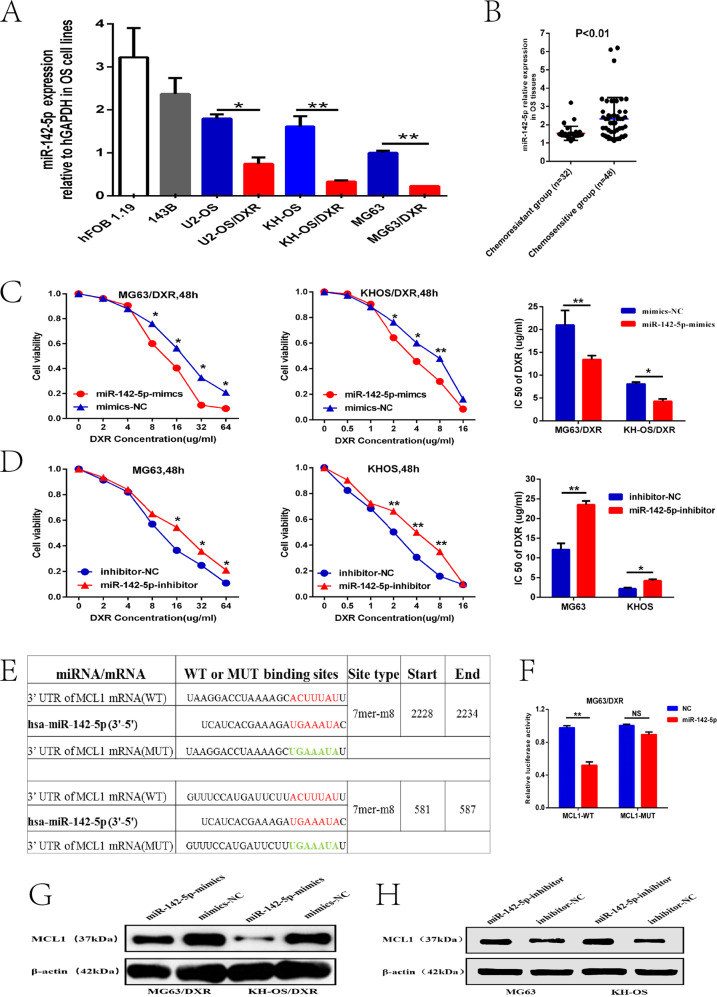
MiR-142-5p suppressed OS chemoresistance by targeting MCL1. **A** miR-142-5p expression level was downregulated in the three chemoresistant OS cell lines and 143B compared to hFOB1.19. **B** miR-142-5p level was dramatically reduced in chemoresistant OS tissues compared to the control group (1.5 ± 0.5 vs. 2.3 ± 1.3). **C, D** CCK-8 assays showed that cell resistance to doxorubicin was obviously increased in the miR-142-5p inhibitor group but was obviously decreased in the miR-142-5p mimics group compared to the NC group. **E** WT and MUT sequences designed for the 3’ UTR of MCL1 mRNA according to its putative binding sites with miR-142-5p. **F** The luciferase activity assay showed that MCL1 could combine with miR-142-5p. **G** The protein expression of MCL1 was significantly declined in the miR-142-5p mimics group compared with the NC group of MG63/DXR (or KH-OS/DXR) cell lines. **H** The protein expression of MCL1 was obviously increased in the miR-142-5p inhibitor group compared with in the NC group of MG63 (or KH-OS) cell lines. *P < 0.05, **P < 0.01.

A previous study reported that miR-142-5p can suppress proliferation and promote apoptosis of OS cell line through the ERK1/2 signaling pathway by targeting PLA2G16 [17]. Nevertheless, the function of miR-142-5p in OS chemoresistance has not been identified. We then examined miR-142-5p levels in the previously described OS chemoresistant and chemosensitive tissues and cell lines, and found that miR-142-5p was dramatically reduced in the chemoresistant OS cell lines and tissues compared with the control group (Fig. 4A, B). MG63/DXR (or KH-OS/DXR) cells stably transfected with the miR-142-5p mimicss and MG63 (or KH-OS) cells transfected with miR-142-5p inhibitor were established. The CCK-8 assay showed that OS cell resistance to doxorubicin was obviously increased in the miR-142-5p inhibitor group and was obviously decreased in the miR-142-5p mimics group compared to the control group (Fig. 4C, D).

As Li et al. previously reported that miR-142-5p targeted MCL1 and other multiple antiapoptotic genes, and enhanced cisplatin-induced apoptosis in ovarian cancer cells [18]; and Su J et al. showed that NF1 could target MCL1 via miR-142-5p to regulate apoptosis in ovarian cancer cells [19]; so we speculated that miR-142-5p could regulate OS chemoresistance by affecting the expression of the antiapoptotic protein, MCL1, a member of the Bcl-2 family. The potential binding sites between the miR-142-5p and 3’UTR of MCL1 mRNA were predicted by the TargetScan and MiRanda databases, and the corresponding mutant sequences were designed **(**Fig. 4E). A dual-luciferase reporter assay further demonstrated that co-transfection of the luciferase reporter plasmid containing WT-MCL1 and the miR-142-5p mimicss into MG63/DXR cells resulted in declined reporter activity compared with the MUT-MCL1 group (Fig. 4F). Moreover, the protein level of MCL1 was significantly declined in the miR-142-5p mimics group compared with the NC group of MG63/DXR or KH-OS/DXR cell lines, whereas it was obviously increased in the miR-142-5p inhibitor group compared with the NC group of MG63 or KH-OS cell lines (Fig. 4G, H). These results indicated that miR-142-5p may inhibit OS chemoresistance by directly targeting MCL1.

### Circ_0004674 regulated the expression of MCL1 through competitive binding with miR-142-5p

**Fig. 5:**
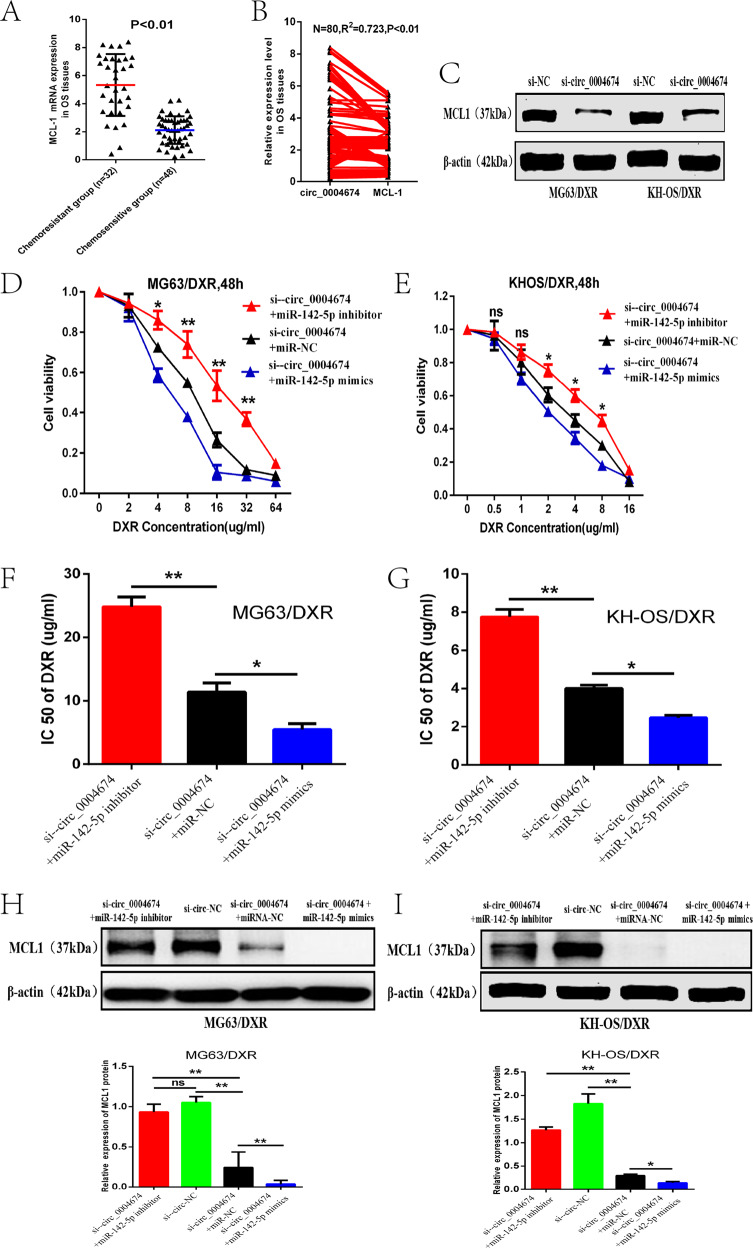
Circ_0004674 regulated the expression of MCL1 through competitive binding with miR-142-5p. **A**The MCL1 mRNA expression level examined by qPCR was higher in the chemoresistant OS tissues than in the control group (5.5 ± 2.2 vs. 2.0 ± 0.9). **B** The expression of MCL1 mRNA was positively correlated with the expression of circRNA_0004674 in the OS tissues. **C** MCL1 protein expression was significantly decreased when knockdown the expression of circRNA_0004674 in the MG63/DXR (or KH-OS/DXR) cell lines transfected with siRNA. **D, E** Downregulation of circRNA_0004674 by siRNA reduced the viability of MG63/DXR(or KH-OS/DXR) cells after exposure to doxorubicin, which was partly reversed after co-transfection with miR-142-5p inhibitor and further reduced by co-transfection with miR-142-5p mimicss. **F, G** The IC50 value of MG63/DXR (or KH-OS/DXR) cells significantly increased in the group transfected with si-circ_0004674 and miR-142-5p inhibitor and greatly decreased in the group transfected with the si-circ_0004674 and miR-142-5p mimicss compared with the control group. **H, I** The protein expression of MCL1 was slightly decreased in the group transfected with si-circ_0004674 and the miR-142-5p inhibitor and distinctly inhibited in the group transfected with the si-circ_0004674 and miR-142-5p mimicss compared with the control group. *P < 0.05, **P < 0.01.

To further identify the relationship between circ_0004674 and MCL1, we also examined the MCL1 expression in the 80 OS tissues previously described and found that just like circRNA_0004674, the MCL1 mRNA expression level was higher in the chemoresistant group than in the control group (Fig. 5A). Besides, we also found that the expression of MCL1 mRNA was positively correlated to the expression of circRNA_0004674 in the OS tissues (Fig. 5B). Moreover, we found that MCL1 protein expression was significantly decreased when knockdown the expression of circRNA_0004674 by siRNA in the MG63/DXR (or KH-OS/DXR), which may suggest the potentially regulatory correlation between them (Fig. 5C). To demonstrate the inner interaction among the three molecules, as shown in the figure, co-transfection of the miR-142-5p inhibitor and si-circ_0004674 showed that miR-142-5p inhibitor partly helped MG63/DXR (or KH-OS/DXR) cells regain the resistance to doxorubicin that were suppressed by circ_0004674 knockdown. Meanwhile, co-transfection of miR-142-5p mimics and si-circ_0004674 demonstrated that miR-142-5p further lowered the doxorubicin resistance of MG63/DXR (or KH-OS/DXR) cells which were suppressed by circ_0004674 knockdown, and this may suggest the antagonistic role of regulation in OS chemoresistance between circ_0004674 and miR-142-5p (Fig. 5D–G). In addition, MCL1 protein expression was distinctly inhibited in the si-circ_0004674 combined with the miR-142-5p mimics group and was obviously upregulated in the si-circ_0004674 combined with the miR-142-5p inhibitor group compared with those in the control group, which uncovered that the expression of MCL1 was co-controlled by circ_0004674 and miR-142-5p (Fig. 5H and I).

## Discussion

Recent researches have showed that circRNAs could take a crucial part in the initiation and development of various cancers, such as lung adenocarcinoma, hepatic carcinoma, prostate cancer, and osteosarcoma [20]. Underlying the potential molecular mechanism of circRNAs in OS progression may be novel promising therapeutic targets. Several circRNAs have been reported to participate in OS tumorigenesis and progression. For example, Wang Z et al. reported that circ-03955 promotes the epithelial-mesenchymal transition (EMT) in OS by regulating the miR-3662/metadherin pathway [21]. Jiang X et al. found that circ_0000658 inhibits OS cell proliferation and migration via the miR-1227/IRF2 axis [22]. Ji X et al. showed that circ_001621 can sponge miR-578 and regulate VEGF expression and thus promote OS cell proliferation and migration [23].

In our previous study, we first identified and reported the comprehensive differentially expressed circRNA expression profile and its related ceRNA regulatory network involved in OS chemoresistance [15, 24]. Of all the screened dysregulated circRNAs, hsa_circ_0004674, the research object in the current study, was the most upregulated with a 17-fold change in the OS chemoresistant cell lines. Here, we further found that circ_0004674 was highly expression in chemoresistant OS tissues and cell lines. Besides, the high circ_0004674 expression was related to OS patients’ poor prognosis, and high circ_0004674 expression was indicated as an independent prognostic factor for OS patients. Subsequently, this study further investigated biological functions of circ_0004674 in OS. Results showed that knockdown of circ_0004674 inhibited OS cell resistance to doxorubicin, proliferation, migration, invasion, tumor growth, and promoted cell cycle arrest and apoptosis in vitro and in vivo.

It has been reported that circRNAs could control gene expression at different levels including pretranscription, transcriptional and post-transcriptional, and this function is strongly correlated with their subcellular location [25]. CircRNAs locate in the nuclei often function at the level of pretranscription or transcription, whereas cytoplasmic circRNAs often function as ceRNAs, and regulate the expression of targeted mRNAs at the post-transcriptional level [8]. In this study, we found that circ_0004674 mainly located in the cytoplasm, as verified by RNA FISH, which may indicate that circ_0004674 could perform its regulatory function at the post-transcriptional level. Then, the ceRNA mechanism was primarily considered. Several circRNAs have been found to take an important part in tumorigenesis and the progression of many tumors by sponging miRNAs to counteract miRNA-mediated repression of mRNA. For example, Sang Y et al. found that circRNA_0025202 regulates tamoxifen sensitivity and tumor progression by regulating the miR-182-5p/FOXO3a axis in breast cancer [26]. Chen L et al. showed that circular RNA 100146 functions as an oncogene in non-small cell lung cancer through direct binding to miR-361-3p and miR-615-5p [27]. Bian L et al. reported that circRNA_103809 regulated colorectal cancer cell proliferation and migration through the miR-532-3p/FOXO4 axis [28]. In this study, we found circ_0004674 could promote OS chemoresistance and progression for the first time. Considering the negative correlation between circRNA and miRNA expression in the ceRNA network, further bioinformatics analysis based on three common databases was performed to seek the downregulated miRNAs which have potential binding sites with circ_0004674 in OS. Two miRNAs were predicted, and one of them, miR-142-5p, was further confirmed that it could directly target circ_0004674 and the antiapoptotic MCL1 gene in MG63/DXR and KH-OS/DXR cells.

There have been several reports on miR-142-5p and OS or chemoresistance. Zhu W et al. reported that miR-142-5p reversed resistance to gefitinib of lung cancer cells by targeting HOXD8 [29]. Li X et al. found that miR-142-5p enhances cisplatin-induced apoptosis in ovarian cancer cells through targeting multiple antiapoptotic genes [18]. Klümper T et al. showed that expression differences of miR-142-5p between treatment-naive chronic myeloid leukemia patients responding and nonresponding to imatinib therapy suggest a link to oncogenic ABL2, SRI, cKIT, and MCL1 signaling pathways that are critical for the development of therapy resistance [30]. In addition, miR-142-5p has been reported to suppresses proliferation and promotes apoptosis in OS cell line by the ERK1/2 signaling pathway [17]. Kevin B Jones et al. showed that miR-142-5p was related to the pathogenesis and progression of OS [31]. The current study also revealed that miR-142-5p was remarkably low expression in chemoresistant OS tissues and cell lines. Furthermore, functional assays showed that miR-142-5p inhibited OS chemoresistance in vitro. In addition, knockdown of miR-142-5p rescued the effect of si-circ_0004674 on OS cell chemoresistance and the expression of MCL1, which may confirm the antagonistic role of regulation in OS cell chemoresistance and MCL1 expression between circ_0004674 and miR-142-5p. But miR-142-5p mimics did not show the resembled effect same with si-circRNA_0004674 in the KHOS/DXR cells, suggesting that other factors may contribute. In fact, one circRNA usually has many potential binding sites with miRNA or RNA binding protein (RBP), which indicate that circRNA could regulate the gene expression through many different miRNAs and (or) RBPs. As for the current shown results, we speculated that there are probably some other miRNA-mRNA pairs regulated by the circRNA_00 04674, which may also involve in the regulation of OS chemoresistance.

Moreover, the expression of MCL1 mRNA was found to be a positive relationship with the expression of circRNA_0004674 in the OS tissues and MCL1 protein expression was significantly decreased when knockdown the expression of circRNA_0004674 by siRNA in the MG63/DXR (or KH-OS/DXR). What’s more, the direct binding of circ_0004674 and the 3′UTR of MCL1 mRNA with miR-142-5p was further verified by the dual-luciferase reporter gene assay, RIP, and biotin-coupled probe pull-down assay. Taken together, these results showed that circ_0004674 could effectively sponge miR-142-5p to promote OS chemoresistance and progression by upregulating the expression of the antiapoptotic protein, MCL1. However, there were still some limitations of the current study. Owing to the failure of overexpression of circ_0004674 in OS cells, it is difficult to identify its oncogene role from the pros and cons in vitro and in vivo.

To sum up, we identified that highly expressed circ_0004674 could act as an oncogene and might serve as a prognostic biological marker in OS chemoresistance and progression. In addition, our study sheds light on the role of the doxorubicin-induced circ_0004674/ miR-142-5p/MCL1 pathway in OS and discloses that circ_0004674 promotes OS chemoresistance and progression by sponging miR-142-5p and targeting antiapoptotic protein MCL1, a member of the Bcl-2 family, thus likely providing a novel potential target for OS treatment.

## Methods and materials

### Cell lines and culture conditions

Human OS cell lines (MG63, KH-OS, U2-OS, 143B) were purchased from American Type Culture Collection (ATCC) and cultured in Dulbecco’s modified Eagle’s medium (DMEM) supplemented with 10% fetal bovine serum (FBS) (Gibco, NY, US A), 100 U/ml penicillin and 0.1 mg/ml streptomycin (Invitrogen, CA, USA) at 37 °C in a humidified CO_2_ (5%) atmosphere. Normal osteoblast cells (hFOB1.19) obtained from the Chinese Cell Bank of the Chinese Academy of Sciences (Shanghai, China) were cultured in Ham’s F12/DMEM supplemented with 10% FBS, 100 U/mL penicillin, 0.1 mg/mL streptomycin, and 0.3 mg/mL G418. Cultures were maintained at 33.5 °C in a humidified CO_2_ (5%) atmosphere. The three paired DXR-resistant OS cell lines (MG63/DXR, KH-OS/DXR, and U2-OS/DXR) were kindly donated by Dr. Oda Yoshio, Dr. Z.F. Duan, and Dr. E.S. Gonos, respectively, which has been described in our previous study [24].

### Patient samples

A total of 80 primary OS patients who were enrolled in the study were also described in our previous study [32], which was approved by the Ethics Committee of Shanghai Tenth People’s Hospital, and written informed consent was obtained from all the patients. The patients were classified into chemoresistant and chemosensitive groups based on the Huvos scoring system. The patients’ characteristics in this study are summarized in Table 1.

**Table 1:** Clinical parameters of osteosarcoma patients enrolled in this study.

Pathological characteristics	Cases (n)	hsa_circ_0004674 expression		*P* value
		High (40)	Low (40)	
**Gender**				
Male	47 (58.8%)	24 (60.0%)	23 (57.5%)	NS
Female	33 (41.2%)	16 (40.0%)	17 (42.5%)	
**Age**				
≥25	24 (30%)	11 (27.5%)	13 (32.5%)	NS
<25	56 (70%)	29 (72.5%)	27 (67.5%)	
**Location**				NS
Distal of Femur	37 (46.3%)	18 (45.0%)	19 (47.5%)	
Proximal of Tibia	27 (33.8%)	15 (37.5%)	12 (30.0%)	
Other	16 (19.9%)	7 (17.5%)	9 (22.5%)	
***Enneking*** **stage**				<0.01
I + IIA	23 (28.8%)	6 (15.0%)	17 (42.5%)	
IIB/III	57 (71.2%)	34 (85.0%)	23 (57.5%)	
**Chemoresistant**				<0.01
Yes	32 (40%)	21 (52.5%)	11 (27.5%)	
No	48 (60%)	19 (47.5%)	29 (72.5%)	

### RNA extraction and qRT-PCR

Total RNA was isolated from OS cells and tissues using TRIzol reagent (Invitrogen). RNA was reverse transcribed to cDNA using the PrimeScript™ RT reagent Kit with gDNA Eraser (Takara, Japan) and amplified by qRT-PCR with a SYBR Green Kit (Takara Bio Company) on an ABIPRISM 7500 Sequence Detection System (Applied Biosystems, Foster City, CA) with the housekeeping gene GAPDH as an internal control. All the primers were synthesized by Sangon Biotech (Shanghai, China), and the primer sequences are shown as follows: circ_0004674 forward primers: 5′-GTTGACCAAGCAAGCTTCCAG-3′; reverse primers: 5′-GGTACTTGCAGGT TTTACTGGG-3′; ADAM22 forward primers: 5′-GGACCTCACAGTCACGAGG T-3′; reverse primers: 5′-TCAGTGCTGCATTGTGCTTC-3′; GAPDH forward primers: 5′-TCTCTGCTCCTCCTGTTCGA-3′; reverse primers: 5′-GCGCCCAATA CGACCAAATC-3′; U6 forward primers: 5′-CAGCACATATACTAAAATTGGAACG-3′; reverse primers: 5′- ACGAATTTGCGTGTCATCC-3′.

### RNase R treatment and Actinomycin D

Total RNA of circ_0004674 and ADAM22 (10 μg) was incubated with RNase R (R0301, Geneseed, Guangzhou, China) at 37 °C for 60 min to analyze the RNase R resistance. Actinomycin D (2 mg/mL, 129935, Millipore, USA) was added to the cell culture medium, and the half-lives of circ_0004674 and ADAM22 were evaluated and analyzed. After treatment with actinomycin D or RNase R, the expression levels of circ_0004674 and ADAM22 were detected by qRT-PCR.

### Plasmid construction and cell transfection

MG63/DXR and KH-OS/DXR cells were transiently transfected with siRNAs (or miR-142-5p mimicss, miR-142-5p inhibitor, miR-NC) using Lipofectamine 2000 transfection reagent (Invitrogen, Carlsbad, CA) according to the manufacturer’s instructions after being seeded into 6-well plates overnight. Forty-eight hours after transfection, the cells were harvested to detect the knockout efficiency via qRT-PCR detection of the expression of circ_0004674 and ADAM22. Two different siRNAs against the junction site of circ_0004674 were designed and synthesized by GenePharma (Shanghai, China). The sequence of si-circ_0004674-1 was 5′-GTCGTGCTAAATCAATTCAGA-3′, that of si-circ_0004674-2 was 5′-GTGCTAAA TCAATTCAGATGT-3′ (si-circ_0004674-1 has the highest inhibition efficiency, and si-circ_0004674 mentioned in the article refers to si-circ_0004674-1) and the relative si-NC sequence was 5′-AAUUCUCCGAACGUGUCACGU-3′.

### CCK-8 assay

Stably transfected MG63/DXR (or KH-OS/DXR) cells (5 × 10^3^ cell/well) were seeded in 96-well plates, freshly prepared medium containing several final concentrations of doxorubicin (0, 2, 4, 8, 16, 32, and 64 μg/ml) was added to the wells with three replicate wells for each concentration. After incubation for another 48 h, cell viability was measured using Cell Counting Kit-8 (CCK-8, Dojindo, Japan) according to the manufacturer’s instructions. The absorbance of each well was measured with a microplate reader set at 450 nm.

### Flow cytometry

For the cell cycle assay, after 48 h of transfection, the cells were digested with trypsin, and the supernatant was discarded. Subsequently, the cells were washed with PBS, centrifuged, and fixed in 75% ethanol at 4 °C overnight. After washing twice with PBS, the cells were stained with propidium iodide (PI) at room temperature in the dark for 15 min before analysis.

For the cell apoptosis assay, after 48 h of transfection, doxorubicin (8 μg/ml) was added to the wells. After incubation for another 12 h, the Annexin V-FITC Apoptosis Detection Kit (BD Biosciences, San Diego, USA) was used to stain cells according to the manufacturer’s protocol. The cell cycle and apoptosis were then analyzed by FACS scan flow cytometry using FlowJo software (BD Biosciences).

### Transwell assay

For the invasion assays, a 24-well Transwell chamber with the upper chamber coated with Matrigel (354230, BD, USA) was used. A total of 5.0 × 10^4^ stably transfected MG63/DXR (or KH-OS/DXR) cells in 200 µL of serum-free DMEM were seeded in the upper chamber, and 600 µl of medium containing 10% FBS was placed in the lower chamber. After incubation for 24 h, cells on the upper membrane surface were wiped off using a cotton swab, and the invading cells that had traversed the membrane were stained with crystal violet and counted.

### Wound healing assay

A total of stably transfected MG63/DXR (or KH-OS/DXR) cells (5 × 10^5^ cell/well) were seeded in 6-well plates. Wounds were then created on a monolayer of cells using a sterile pipette tip. Cells were further cultured with a medium containing 1% FBS for 48 h. The healing wounds were photographed twice at 0 h and 48 h after scratching, and then, the cell migration rate was calculated.

### Xenograft tumor assay

Female nude (BALB/c) mice (4 weeks old) were purchased. Mice were divided into two groups according to the completely randomized method (*N* = 6/group). All procedures for the mouse experiments were approved by the Animal Experimental Ethics Committee of Shanghai Tenth People’s Hospital. MG63/DXR cells stably expressing sh-circ_0004674 or sh-NC were propagated, and 5 × 10^6^ (100 µl) cells were inoculated into the medullary cavity of the right proximal tibia of mice. Tumor growth was examined at the indicated time points, and tumor volumes were measured. After 7 weeks, the mice were killed, and tumors were removed and weighed. The activity of Ki-67 and caspase3 in the tumors was measured by immunochemistry (IHC).

### Bioinformatics prediction

The sequence of circ_0004674 was obtained from Circbase (http://www.circbase. org/cgi-bin/simplesearch.cgi), and visual graphics of circ_0004674 were obtained from CircPrimer, a software which is for annotating circRNAs and determining the specificity of circRNA primers. CircInteractome (https://circinteractome.nia.nih.gov/) was used to analyze the target miRNA of circ_0004674. All the downregulated miRNAs in OS previously reported were collected from the database of dbDEMC (database of Differentially Expressed MiRNAs in human Cancers, https://www.picb. ac.cn/dbDEMC/index.html) and miRCancer (microRNA Cancer Association Database, http://mircancer.ecu.edu/).

### RNA immunoprecipitation (RIP)

RIP experiments were performed in MG63/DXR cells using an RIP Kit (Millipore, Billerica, MA) following its manufacturer’s protocol. Cells were lysed and incubated with RIP buffer containing A/G magnetic beads conjugated with Ago2 antibody (Abcam) and normal anti-IgG (Millipore) as a negative control. Finally, immunoprecipitated RNA was isolated and purified for qRT-PCR to analyze the expression levels.

### Biotin-coupled probe pull-down assay

Biotin-coupled probe pull-down assays were performed to determine the interaction between circ_0004674 and miR-142-5p. Briefly, the miR-142-5p-WT or miR-142-5p- Mut probe was synthesized and biotinylated by GenePharma (Shanghai, China). RNA pull-down assays were carried out using the Magnetic RNA-Protein Pull-Down Kit (Thermo Fisher Scientific, Waltham, MA) following its manufacturer’s protocols. Finally, the RNA binding protein complexes were washed and eluted for qPCR analysis of circ_0004674.

### Dual-luciferase reporter assay

MG63/DXR cells were seeded at 5 × 10^4^ cells/well in 24-well plates and allowed to settle overnight. The next day, cells were co-transfected with pmirGLO-circ_0004674 (or MCL1)-WT or-MUT reporter plasmids and miR-142-5p mimics. Twenty-four hours after transfection, 20 µl of the protein supernatant was added to 50 µl of the firefly luciferase substrate and mixed thoroughly. Then, the relative luciferase activity was measured using the Dual-Luciferase Reporter Assay System (Promega, Madison, WI, USA) and normalized against Renilla luciferase activity.

### RNA fluorescence in situ hybridization (FISH)

Cy3-labeled circ_0004674, Cy5-labeled miR-142-5p, and DAPI-labeled U6 probes were obtained from GenePharma (Shanghai, China). RNA FISH was performed using a fluorescent in situ hybridization kit according to the manufacturer’s protocol (Thermo Fisher).

### Western blot

For western blotting, the total protein was extracted using RIPA lysis buffer from cells (P0013, Beyotime, CA). Then, the protein loading buffer was added to the protein and mixed, and the mixture was boiled for 10 min. Next, a 30-μg protein sample was added to each well, electrophoresed, and subsequently transferred to a NC membrane (Immobilon-P Transfer Membrane, EMD Millipore Corporation, MA). The membrane was blocked with 5% skim milk for 2 h and then incubated with the primary antibody overnight at 4 °C, followed by incubation with the corresponding secondary antibody for 2 h at room temperature. The protein bands were visualized using an Odyssey scanner (LI-COR Biosciences, Lincoln, NE, USA). ImageJ software was used for semiquantitative analysis. The primary antibodies were MCL1 and β-actin.

### Statistical analysis

All statistical analyses were performed using SPSS 22.0 software (IBM) and GraphPad Prism 7.0 (GraphPad Software Inc, San Diego, CA, USA) software. All the experiments in the current study were independently performed at least three times and data are presented as the means ± SEM. Student’s *t* test or one-way ANOVA was used to evaluate the differences between groups for continuous data. Chi-square test was used to compare the distribution of stage between high- and low-expression circ_0004674 level groups for categorical data. Overall survival was calculated by Kaplan–Meier survival analysis and compared using the log-rank test. The correlation of circ_0004674 and MCL1 mRNA expression level in OS tissues was determined by Spearman’s correlation. Then, *p* values of <0.05 were considered statistically significant.

## Supplementary information

PRE_Authorshipform-Signed section 6

Corrected-PRE_Authorshipform

AJE Editing Certificate

cddiscovery-author-contribution-form

## Data Availability

All data generated or analyzed during this study are included in this published article.
