# Supplementary figures and images for: Doxorubicin-induced novel circRNA_0004674 facilitates osteosarcoma progression and chemoresistance by upregulating MCL1 through miR-142-5p
Source: Cell Death Discov. 2021 Oct 23;7:309. doi: 10.1038/s41420-021-00694-8 (PMC8542045; doi:10.1038/s41420-021-00694-8)

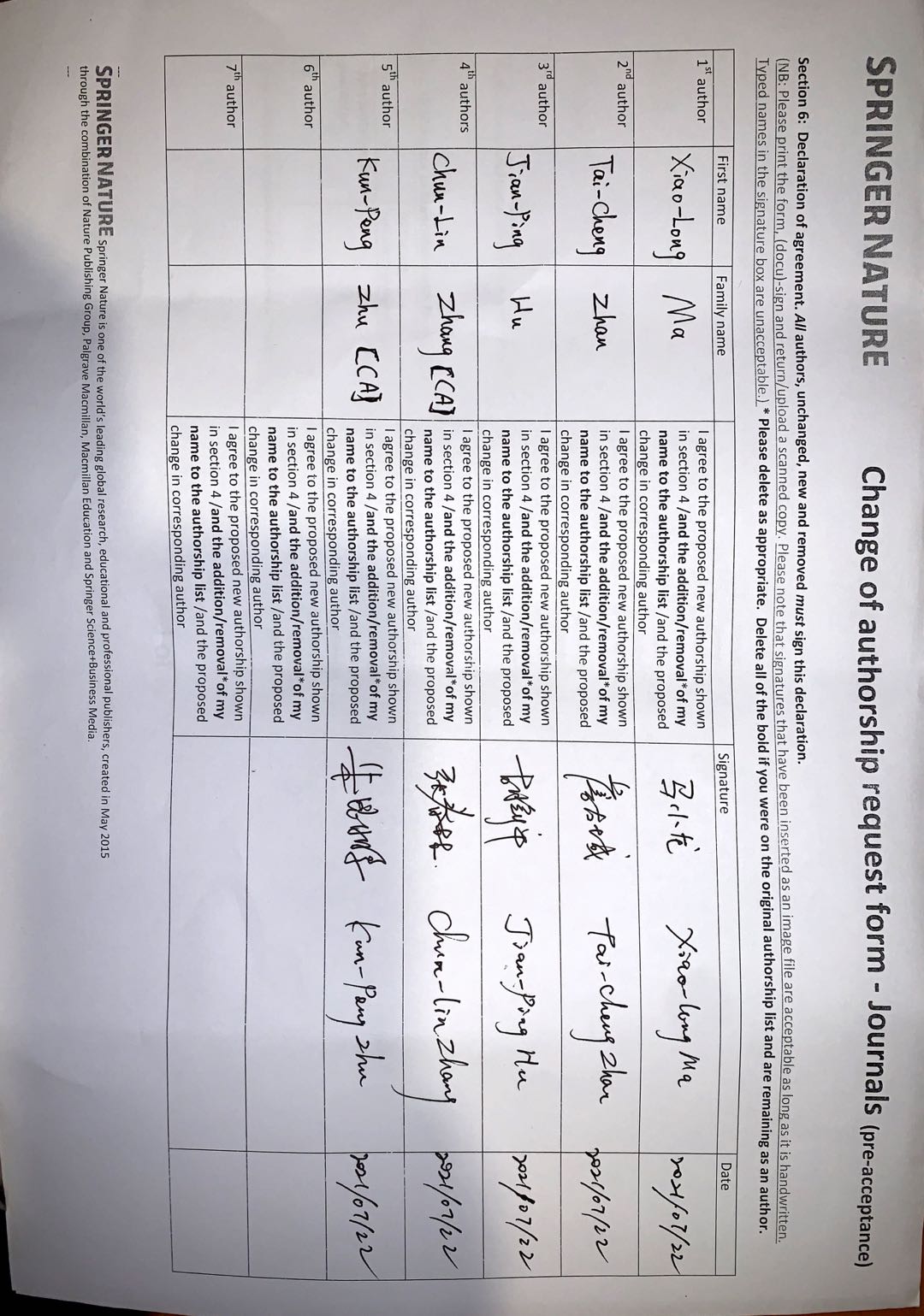

Supplement: Supplementary file 1 — PRE_Authorshipform-Signed section 6 [file 41420_2021_694_MOESM1_ESM.jpg]
