# Supplementary material for: Doxorubicin-induced novel circRNA_0004674 facilitates osteosarcoma progression and chemoresistance by upregulating MCL1 through miR-142-5p
Source: Cell Death Discov. 2021 Oct 23;7:309. doi: 10.1038/s41420-021-00694-8 (PMC8542045; doi:10.1038/s41420-021-00694-8)
Supplement: Supplementary file 3 — AJE Editing Certificate [file 41420_2021_694_MOESM3_ESM.pdf]

Editing Certificate

This document certifies that the manuscript

Doxorubicin-induced novel circRNA_0004674 facilitates osteosarcoma progression and chemoresistance by upregulating MCL1 through miR-142-5p

prepared by the authors

Xiao-Long Ma, Tai-Cheng Zhan, Chun-Lin Zhang, Kun-Peng Zhu

was edited for proper English language, grammar, punctuation, spelling, and overall style by one or more of the highly qualified native English speaking editors at AJE.

This certificate was issued on **July 9, 2021** and may be verified on the AJE website using the verification code **013E-8431-CD69-6626-31FD**.

Neither the research content nor the authors' intentions were altered in any way during the editing process. Documents receiving this certification should be English-ready for publication; however, the author has the ability to accept or reject our suggestions and changes. To verify the final AJE edited version, please visit our verification page at aje.com/certificate. If you have any questions or concerns about this edited document, please contact AJE at support@aje.com.

AJE provides a range of editing, translation, and manuscript services for researchers and publishers around the world. For more information about our company, services, and partner discounts, please visit aje.com.
