# Supplementary material for: Doxorubicin-induced novel circRNA_0004674 facilitates osteosarcoma progression and chemoresistance by upregulating MCL1 through miR-142-5p
Source: Cell Death Discov. 2021 Oct 23;7:309. doi: 10.1038/s41420-021-00694-8 (PMC8542045; doi:10.1038/s41420-021-00694-8)
Supplement: Supplementary file 4 — cddiscovery-author-contribution-form [file 41420_2021_694_MOESM4_ESM.pdf]

DECLARATION OF CONTRIBUTIONS TO ARTICLE

ADMC

Manuscript Number:	Journal Name:	
CDDISCOVERY-21-1726	*Cell Death Discovery*	(the 'Journal')

Proposed Title of the Contribution:

Doxorubicin-induced novel circRNA_0004674 facilitates osteosarcoma progression and chemoresistance by upregulating MCL1 through miR-142-5p (the 'Contribution)

Author(s):

Xiao-Long Ma#, Tai-Cheng Zhan#, Jian-Ping Hu, Chun-Lin Zhang*, Kun-Peng Zhu* (the 'Authors')

For all *CDDis*covery articles, each person named as an author in the published version must be able to show he or she has contributed substantially to the article.

Authorship credit should be based on 1) substantial contributions to conception and design, acquisition of data, or analysis and interpretation of data; 2) drafting the article or revising it critically for important intellectual content; and 3) final approval of the version to be published. Authors should meet conditions 1, 2 and 3.

Any person who cannot be shown to have made a substantial contribution to the article cannot be listed as an author in the final version. The name of any person who is deemed to have made a minor contribution can, however, appear in the Acknowledgments section of the article.

Please complete the table below to indicate the contributions of all named authors to the manuscript.

Author Full Name:	Specification of Contribution to the Manuscript:
Xiao-Long Ma	partly carried out the molecular genetic studies and the tumor-bearing nude mice assays
Tai-Cheng Zhan	carried out the tissue samples collection and statistical analysis.
Jian-Ping Hu	partly carried out the tumor-bearing nude mice assays
Chun-Lin Zhang	participated in the design of the study and helped to correct the manuscript
Kun-Peng Zhu	participated in the design of the study, performed the statistical analysis and drafted the manuscript

Please complete the table below to indicate the contributions of all named authors to the figures.

Figure 1:

Xiao-Long Ma, Tai-Cheng Zhan, Kun-Peng Zhu, Chun-Lin Zhang

Figure 2:

Xiao-Long Ma, Jian-Ping Hu, Kun-Peng Zhu,Chun-Lin Zhang

Figure 3:

Xiao-Long Ma, Kun-Peng Zhu,Chun-Lin Zhang

Figure 4:

Xiao-Long Ma, Tai-Cheng Zhan, Kun-Peng Zhu, Chun-Lin Zhang

Figure 5:

Xiao-Long Ma, Tai-Cheng Zhan, Kun-Peng Zhu, Chun-Lin Zhang

Figure 6:

Signed for and on behalf of the Author(s):	Print Name:	Date:
Kun-Peng Zhu	Kun-Peng Zhu	2021/07/11
